# Insights into the 400 million-year-old eyes of giant sea scorpions (Eurypterida) suggest the structure of Palaeozoic compound eyes

**DOI:** 10.1038/s41598-019-53590-8

**Published:** 2019-11-28

**Authors:** Brigitte Schoenemann, Markus Poschmann, Euan N. K. Clarkson

**Affiliations:** 1 University of Cologne, Zoology Department/ Neurobiology/Animal Physiology/Institute of Biology Education, Herbert-Lewin-Straße 10, D-50931 Cologne, Germany; 2 Generaldirektion Kulturelles Erbe RLP, Direktion Landesarchäologie/Erdgeschichte, Niederberger Höhe 1, D-56077 Koblenz, Germany; 3 University of Edinburgh, Grant Institute, School of Geosciences, West Mains Road, Edinburgh, EH9 3JW Scotland

**Keywords:** Palaeontology, Taxonomy, Visual system, Animal physiology, Marine biology

## Abstract

Sea scorpions (Eurypterida, Chelicerata) of the Lower Devonian (~400 Mya) lived as large, aquatic predators. The structure of modern chelicerate eyes is very different from that of mandibulate compound eyes [Mandibulata: Crustacea and Tracheata (Hexapoda, such as insects, and Myriapoda)]. Here we show that the visual system of Lower Devonian (~400 Mya) eurypterids closely matches that of xiphosurans (Xiphosura, Chelicerata). Modern representatives of this group, the horseshoe crabs (Limulidae), have cuticular lens cylinders and usually also an eccentric cell in their sensory apparatus. This strongly suggests that the xiphosuran/eurypterid compound eye is a plesiomorphic structure with respect to the Chelicerata, and probably ancestral to that of Euchelicerata, including Eurypterida, Arachnida and Xiphosura. This is supported by the fact that some Palaeozoic scorpions also possessed compound eyes similar to those of eurypterids. Accordingly, edge enhancement (lateral inhibition), organised by the eccentric cell, most useful in scattered light-conditions, may be a very old mechanism, while the single-lens system of arachnids is possibly an adaptation to a terrestrial life-style.

## Introduction

**Figure 1:**
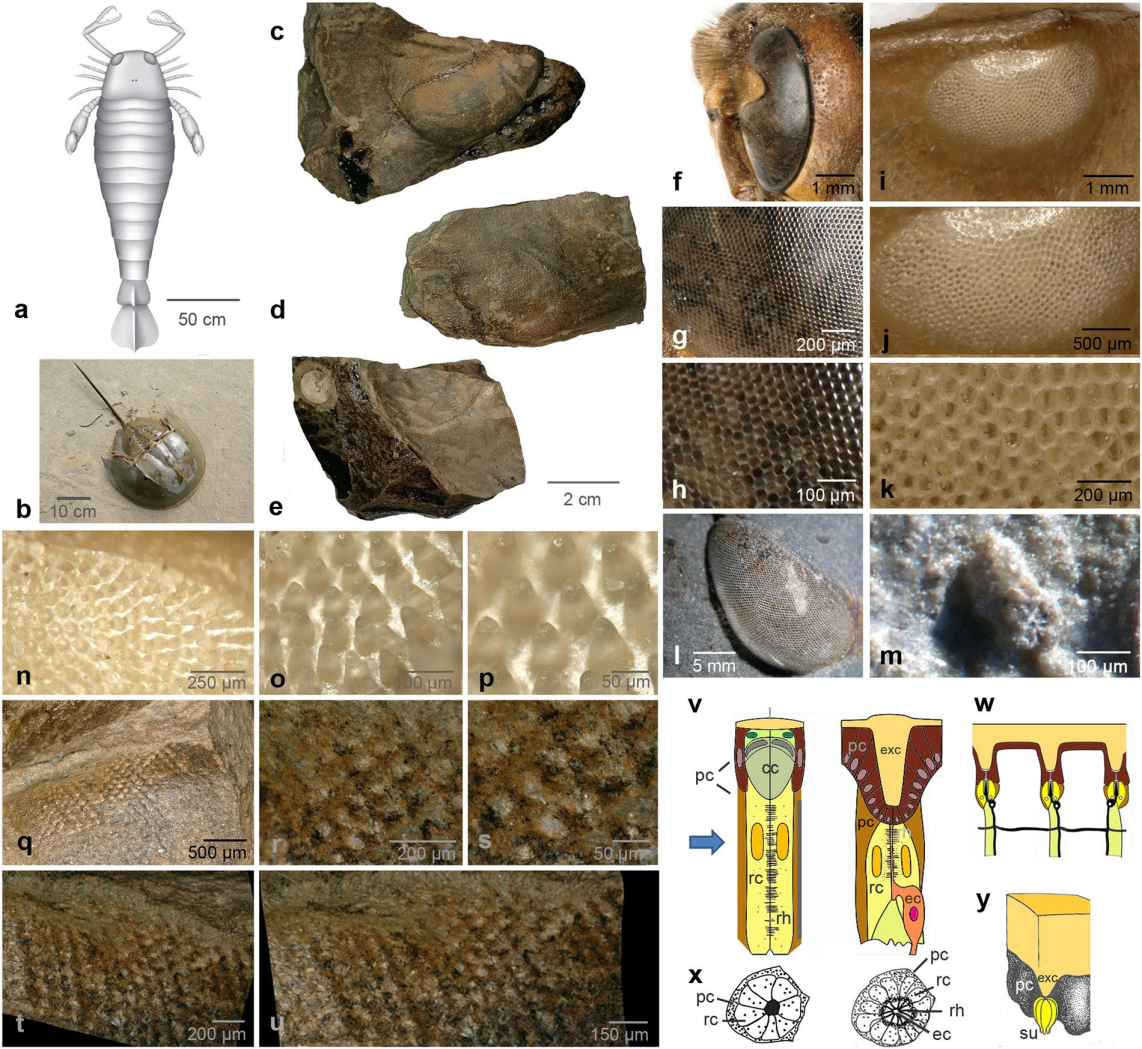
Examples of eurypterid and limulid morphology and comparison of *Jaekelopterus* lateral eyes to other arthropod compound eyes. (**a**) Reconstruction of *Jaekelopterus rhenaniae* (Jaekel, 1914) (modified from Braddy *et al*. 2008; drawing by S. Powell, with permission. (**b**) *Tachypleus gigas* (Müller, 1785), Limulida [© Subham Chatterjee/CC BY-SA 3.0 (via Wikimedia Commons)]. (**c–e**) Examples of specimens (c: GIK 186, d: GIK 188, e: GIK 190). (**f**–**h**) Compound eye of a hornet *Vespa crabro germana* Christ, 1791. (**i–k**) Compound eye of *Carcinoscorpius rotundicauda* (Latreille, 1802), Limulida, exuviae. (**l**) Compound eye of the trilobite *Pricyclopyge binodosa* (Salter, 1859), Ordovician, Šárka formation, Czech Republic. (**m**) Ommatidium of the trilobite *Schmidtiellus reetae* Bergström 1973, base of the Lower Cambrian, Lükati Fm., Estonia. (**n**–**p**) Cones (lens cylinders) of the internal side of the compound eye of *C. rotundicauda* (exuvia). (**q**) Impressions of the exocones in *Jaekelopterus rhenaniae* (Jaekel, 1914), (GIK 186a). (**r**–**t**) Exocones of *J. rhenaniae* (GIK 186), note the regular arrangement compared to (**i**). (**u**) Schematic drawing of the ommatidium of an aquatic mandibulate (crustacean), and of the ommatidium of a limulid. (**w**) schematic drawing of several ommatidia of a limulide. (**x**) Schematic drawing of crossections of (**v**), the limulide after^73^. (**y**) Visual unit of a limulide (redrawn and changed after^51^). cc, crystalline cone; ec, eccentric cell; exc exocone; l, lens; p, pigment; pc, pigment cells; rc, receptor cells; rh, rhabdom; su sensory unit.

Eurypterids, popularly known as sea scorpions, possess conspicuously large compound eyes. Indeed, *Jaekelopterus rhenaniae* (Jaekel, 1914) (Fig. 1a) from the Early Devonian of Germany was perhaps the largest aquatic arthropod ever with a body length approximating 2.5 meters^1^. A fully grown *Jaekelopterus* (Fig. 1a) was a giant even when compared to other large arthropods such as the well-known Cambrian *Anomalocaris* (~1 m long^2^), or the meganeurid griffenflies (~75 cm wing span) of the Permo-Carboniferous^1,3,4^, or *Hibbertopterus*, another eurypterid, which probably was about 1.60 m long^5^. It was only the Late Carboniferous giant millipede *Arthropleura*, which perhaps attained comparable proportions^6^.

Eurypterids appeared during the Middle Ordovician (Darriwilian, 467.3 Mya^7^), but probably originated earlier, and having been already severely affected by the late Devonian environmental changes, they vanished before the end of the Permian (252 Mya)^8^), when up to 95% of all marine species died out^9,10^.

Most eurypterids were predators, as indicated by morphological attributes such as gnathobasic coxae. Some species were equipped with spinose legs and/or highly effective grasping chelicerae or claws. In *J. rhenaniae* these claws reached an impressive size of 46 cm^1^. Forms lacking such specialized morphology, probably nectobenthic, walking on or swimming close to the sea floor. As opportunistic feeders, they dug in the mud as do modern horseshoe crabs, grabbing and shredding whatever they found to eat^11^. While earlier eurypterids were marine, among the later, younger forms some were living in brackish or even fresh water (e.g.^12–15^). In addition to book gills comparable to those of modern *Limulus*, ancillary resiratory organs known as Kiemenplatten^16^ may have allowed the animals to make short visits to the land^17^, but work currently in progress suggest that these may have aided gas exchange in aquatic settings^18^. Some eurypterids were ambush predators, e.g., the large pterygotid *Acutiramus*, based on analyses of functional morphology of its chelicerae^19^ and its visual capabilities^20^. On the other hand, the ecological requirements of eurypterids were apparently quite diverse and results based on analysis of a particular taxon (or species) may not apply to other clades^21,22^.

At least some eurypterids were effective predators, and most of such predators are, and were, equipped with an excellent visual system. Not much, however, has so far been understood about eurypterid eyes. In systematic terms, Tollerton^23^ defined nine different shapes of eurypterid eyes, ranging from small lunar shaped examples to domed ovoid types, of which some even had a frontally overlapping visual field (e.g., *J. rhenaniae*). The latter is typical for predators, allowing stereoscopic vision, which is imperative for the estimation of distances, volumes etc. Assuming that there is an optimal trade-off in compound eyes working at threshold perception between maximal acuity (demanding a high number of facets) and a high sensitivity (requiring large lenses) it has been possible to assign different eurypterid species to their light-ecological environments^20–22^. The results show a transition in lateral eye structure in eurypterids as a whole, and furthermore reflect a niche differentiation in co-occurring (Rhenish) eurypterids, thus avoiding competition for food in their marginal to delta plain habitats^22^.

Nothing, however, has been documented about the internal structure of the eurypterid visual system, which might tell us about function and phylogenetic context.

## Systematic Position of Eurypterida Burmeister, 1843

Soon after the first eurypterid fossils were described, they were regarded as close relatives of xiphosurans^24^ and the two groups were united in the taxon Merostomata Dana, 1852^25^. Traditionally, the major arthropod clade Chelicerata Heymons, 1901 has been divided into the aquatic Merostomata (Xiphosura + Eurypterida) and the terrestrial Arachnida, but today, most arthropod workers consider Merostomata a paraphyletic grade of aquatic chelicerates^26–28^ and the term is no longer used. On the other hand, eurypterids have long been considered as closely allied to arachnids^27–29^, and scorpions have been regarded in some analyses as the closest relatives of eurypterids^30–32^. For further morphological evidence of the close relationship between scorpions and eurypterids, a recent study of cuticle microstructure found similarities between scorpions, eurypterids, and horseshoe crabs^33^. Versluys and Demoll^34^ emphasized the similar body segmentation in scorpions and eurypterids, and Dunlop & Selden^29^ later pointed out that the 5-segmented postabdomen can be regarded as a synapomorphic trait shared by scorpions and eurypterids. Kamenz *et al*.^35^ interpreted organs found in exceptionally well-preserved specimens of *Eurypterus* as structures equivalent to spermatophore-producing organs in the genital tracts of some modern arachnids. In concert with the supposed mating behaviour of eurypterids via transfer of a spermatophore^36^, these findings offered support for a Eurypterida + Arachnida clade, for which the name Sclerophorata has been proposed^34^. Following the Sclerophorata concept, Lamsdell^37^ again supported the view that the eurypterids are more closely related to arachnids than to xiphosurans, which would necessitate their removal from Merostomata *sensu* Woodward^24^ and ultimately renders the latter taxon a junior synonym of Xiphosura. However, the question of whether eurypterids are more closely allied to arachnids or to xiphosurans is still far from settled (see discussion in^11^). Recent phylogenomic analyses of chelicerate relationships even strengthen the concept that the Arachnida are polyphyletic, and also a nested placement of Xiphosura within Arachnida (^38^ and references therein). Thus, understanding the internal structure and function of eurypterid eyes not only sheds light on the origins of chelicerate eyes, but may also offer further insights into the ecology, behaviour, and relationships of these extinct invertebrates.

## The Eyes of Mandibulates, Arachnids and Horseshoe Crabs

The oldest compound eye known at present, is that of the lower Cambrian trilobite *Schmidtiellus reetae* Bergström, 1973 Figs. 1m, 2e), which has a typical apposition compound eye (Fig. 1f–h,m,v,x), not dissimilar to that of modern bees, dragonflies and many crustaceans^39^, and there is strong evidence for a common origin of insect and crustacean eyes^40–42^. These eyes consist of repeated identical visual units, the ommatidia, appearing externally as facets. Each ommatidium contains ~8 receptor cells, grouped around a central axis, the rhabdom. The rhabdom is part of the sensory cells, and contains the visual pigments. With light energy these are changed in their sterical configuration, and a small electrical signal is sent to the nervous system. The incident light is focused onto the tip of this rhabdom by a dioptric apparatus, consisting of a cuticular lens and a cellular crystalline cone, while in modern aquatic visual systems the latter typically takes over the function of refraction. All ommatidia are isolated optically from each other by screening pigment cells, and thus over the whole compound eye a mosaic-like image is formed. In the ancient system of the trilobite *S. reetae*, however, a lens and a crystalline cone are not very evident, and the sensory unit sits in a kind of basket, isolating the ommatidia against each other. Principally, among other factors, the acuity of vision depends on the number of facets, and the apposition eye is typically found in animals active when the light is bright^43^. Adaptations of such eyes to dimmer light conditions, systems such as superposition eyes, are not known before the Devonian^44^.

**Figure 2:**
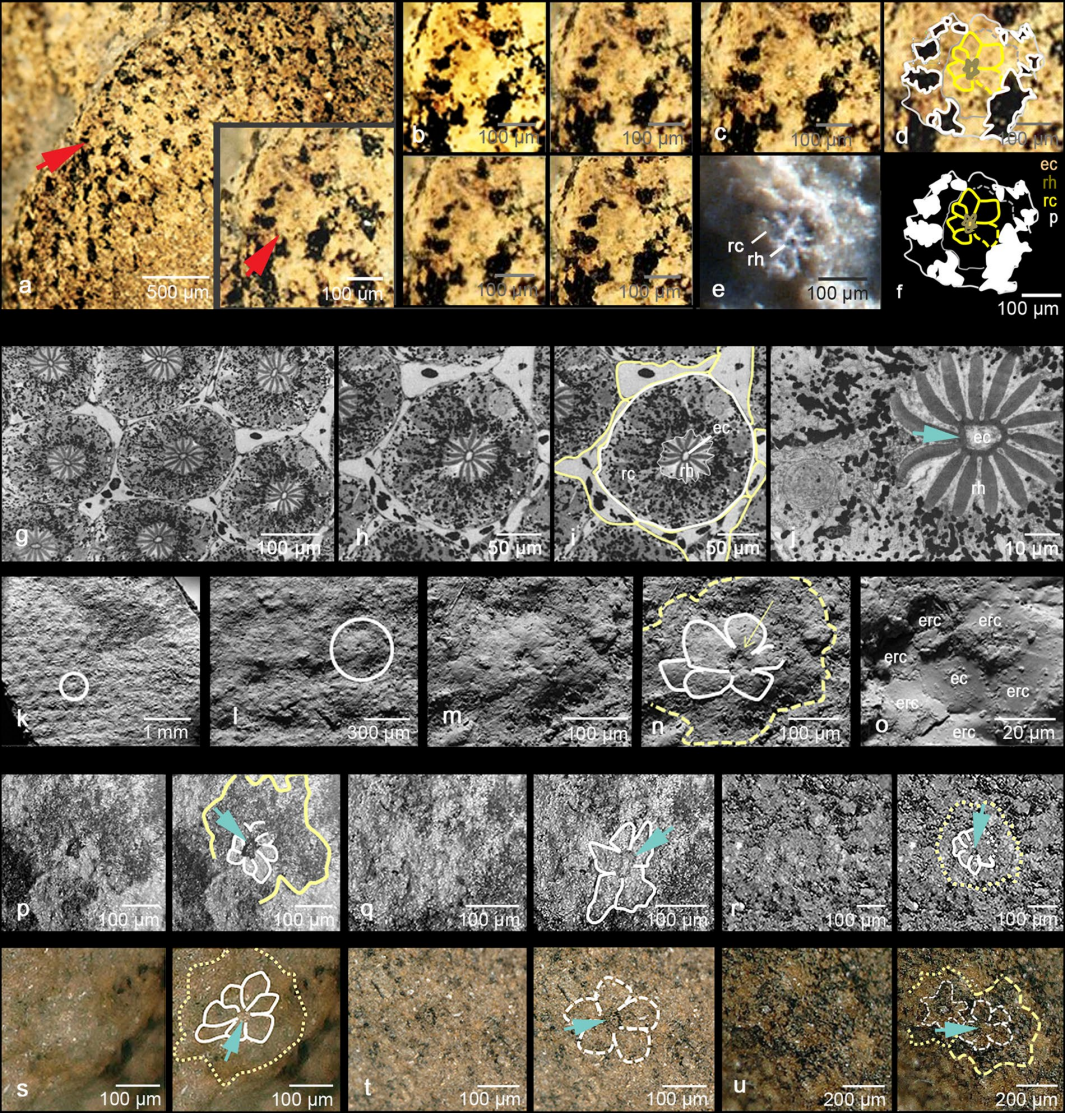
The ommatidium of *Jaekelopterus rhenaniae* (Jaekel, 1914). (**a**–**d**) The specimen, where the first ommatidium was discovered (red arrow), d: brown: rhabdom with eccentric cell dendrite in the centre, yellow: receptor cells, black spots: possibly screening pigments. (**b**) shows the rosette of the ommatidium in crossection under different contrasts. Note the bright spot of the presumed eccentric cell in the centre of the rhabdom. (**c**,**d**,**f**) Rosette of (**a**) magnified, and its schematic drawings. (**e**) Ommatidium of the trilobite *Schmidtiellus reetae* Bergström 1973 and its homogenous rhabdom. (**g**–**j**) Cross sections of ommatidia of *Limulus* (quoted from^69^ Figs. 2 and 3)), [white circle indicates the rosette, yellow circle the pigmental periphery, blue arrow the situation of the rhabdom, (**j**). (**k–o**) SEM of the compound eye of *Jaekelopterus rhenaniae* (Jaekel, 1914), total aspect (**k**) to one rosette (**m**–**o**) and individual rhabdom (**o**), [white circle indicates the rosette of receptor cells, yellow circle the pigmental periphery, yellow arrow the situation of the rhabdom, (**n**)]. (**p–u**) Different sensory rosettes of *J. rhenaniae* (Jaekel, 1914) and their interpretative drawings. Note the bright patch in the centre of the rhabdom, comparable to (**a**–**d**), and (**g**–**j**). (**q–r**) In black and white to show contrasts different from (**s–u**) in colour, [white circle indicates the rosette of receptor cells, yellow circle the pigmental periphery, blue arrow the situation of the rhabdom]. p, q GIK 188; (**r**–**t**), w, u GIK 190; ec eccentric cell; exc exocone, erc, element of receptor cell forming the outer part of the rhabdom; p, pigment cells; rc, receptor cells; rh, rhabdom; su sensory unit. Blue arrows indicate the dark rhabdomeric ring with the relics of the presumed dendrite of the eccentric cell inside.

Differences in the ontogenetic development of the compound eyes in xiphosurans and Pancrustacea/Tetraconata (crustaceans and hexapods including insects) exclude a common origin for both systems; they evolved convergently^45^. The xiphosurans such as *Limulus* are well known as descendants of a bradytelic lineage (evolving at a rate slower than the standard), exhibiting only little morphologic change since the Mesozoic^46^ and with roots that can be traced back in time at least to the Early Ordovician^47^. Even, if horseshoe crabs have changed their ecological requirements numerous times in the course of their evolution^48^, the extant species still seem to retain an ancient visual system. Ommatidial facets of living xiphosurans (Figs. 1b,i–k,n–u,v–y, 2g–j), namely *Limulus*, are up to 320 microns^49^ in diameter (among the largest known in the animal kingdom^49^), make the eye a superb light collector^49^, and shows characteristic differences to those of Tetraconata. The dioptric apparatus of the xiphosurans is formed by a cuticular lens cylinder^50^, while in Tetraconata a cuticular lens focuses the light through a cellular crystalline cone. The latter, in water, takes over, by physical reasons, the function of refraction. The number of receptor cells per ommatidium in *Limulus* varies from 4 to 20, (8 to 15) (^51,52^). As in many apposition compound eyes of tetraconates the rhabdomeres of the receptor cells are fused in the centre. In xiphosurans the retinular cells are electrically coupled to one another, especially by the so-called eccentric cell, which sits at the base of the sensory unit. This configuration is not found in the tetraconate system. The rhabdom, which is built by microvillar arrays along the central part of the receptor cells appears like an asterisk with a central ring occupied by the dendrite of the eccentric cell when viewed along the light path (ref. ^52^, p. 112). The eccentric cell takes over first processings among the receptor cells, and coupled to its neighbours it contributes to processes such as lateral inhibition^53^, enhancing edges and boundaries in the perceived visual image. This is beneficial especially in the scattered light-conditions of the water column. In contrast, the fused tetraconate rhabdom is uniform (Figs. 1m,x, 2e). The visual units of both, Tetraconata and xiphosurans, are embraced by pigment cells, screening them against each other and isolating them optically. Thus the mode of the dioptric apparatus and the structure of the rhabdom is the crucial point here for distinguishing the eurypterid ommatidium, and to confirm that it is an ancient chelicerate system and similar to those of xiphosurans, rather than a tetraconate system, or even of a totally different structure.

In spiders and their terrestrial relatives (e. g., phalangids and extant scorpions) the eyes are not compound eyes but all are of the simple corneal type (ref. ^54^, p. 527, for a detailed review see^55^). True spiders (Araneae) usually have 6–8 eyes, the so-called principal eyes (main eyes), homologous to the mandibulate median eyes and looking forward, being adapted to look at objects nearby; the lateral eyes (secondary eyes) cover the more peripheral fields of view. Based on the rhabdomeric pattern of the retina Paulus^56^ supposed that the scorpion eye has evolved from the facet eye by fusion of corneae, and interpreted the secondary eyes of Arachnida as dispersed and reduced former compound eyes (ref. ^56^, p. 311–313). This idea has been taken up to be investigated with modern molecular methods by Morehouse^57^. Miether & Dunlop^55^ documented compound lateral eyes of fossil scorpions and other arachnids. The Triassic (about 220 Mya) scorpions *Mesophonus opisthophthalmus* Wills, 1947 and *Mesophonus perornatus* Wills, 1910 bear at least 28 and 35 facets respectively, the Late Carboniferous (~309 Mya) scorpion *Kronoscorpio danielsi* Petrunkevitch, 1913 (~309 Mya) shows 25–29 individual facets. Also revealing is a specimen from the Lower Devonian Rhynie Chert of Scotland (~410 Mya, *Palaeocharinus* sp.), which belongs to the Trigonotarbida, an extinct group of spider-like arachnids, and possibly closely related to scorpions^58^. It shows three larger lenses, and a horizontal row between them of 10–11 smaller facets - a system which may indicate a transition from the compound to a single-lens eye. Thus the single-lens eyes of modern arachnids including scorpions appear to be highly derived systems, their ancestral structure, however, remains unclear.

Arthropodean eyes of Cambrian age (541–485 Mya, Palaeozoic) are of the compound type^59,60^ and were found e.g. in radiodonts and in great appendage or megacheiran arthropods. While in a single radiodont eye at least 16,000 hexagonally packed facets may be observed^59^, the pattern of the fossil scorpion eyes is much less regular and the single less numerous. The arrangement resembles those of modern xiphosurans. One may keep in mind that the most optimal packing of numerous originally round elements as a matter of principle is that of a hexagonal arrangement, as evident in honeycombs for example. Thus a hexagonal pattern would be expected in high resolution compound eyes with many facets, while for less acute compound eyes the pattern may become more or less irregular, as can be observed in almost all smaller compound or aggregate eyes. Thus it is not the external pattern that may be crucial for phylogenetic discussion, because it is so constructed for functional reasons. This functional pattern from an evolutionary point of view can be developed very quickly, as the now classic model by Nilsson & Pelger^61^ shows. It is the internal structure, namely the organisation of the dioptric and sensory apparatus that is relevant here, or the organisation of the optical lobes (for this see^60^). In principle, under each facet of a compound eye any kind of structure might reside, any mode of ommatidium, a small retina, a singular receptor cell or anything else. The phylogenetic context, however, gives certain constraints.

Likewise, it has been discussed that the myriapod ocellar single-lens eyes had arisen by disintegration of former compound eyes^62^. Each unit consists of a big lens floored by up to several hundreds of cells^63,64^. Harzsch and colleagues showed that during eye growth in Myriapoda new elements are added to the side of the eye field, extending rows of previously generated optical units, which is suggested to contradict the above assumption^64^. Only one group of Chilopoda, the Scutigeromorpha, shows hexagonal facets and their ommatidia possess cone cells^65^, similar to those of Pancrustacea/Tetraconata (hexapods + crustaceans). Their compound eyes, however, must be considered as secondarily reorganized lateral myriapodan ocelli^62^.

## Results

To decide to which type the eyes of eurypterids belong, one has to consider two questions. The first concerns the structure of the dioptric apparatus. If the eye is constructed similarly to that of an aquatic tetraconate as in marine crustaceans, one would expect a distal thin cuticle, not functioning as a lens, which would be typical just for terrestrial arthropods. Instead one might expect a distinct crystalline cone forming the dioptric apparatus. In contrast, in the xiphosuran compound eye, less ordered than a typical tetraconate compound eye, the dioptric apparatus consists of cuticular, cone-shaped lens-cylinders (exocones), which form a pattern of separated domes, visible if the cuticle is removed from the receptors (Fig. 1n–p).

### Exocones in eurypterid eyes

Figure 1r–u clearly shows the relicts of a eurypterid visual surface, appearing much more regular than that of a xiphosuran (Fig. 1j,k,n–p). The units are regularly arranged in a squared pattern. Specimen GIK 186 clearly shows the lower surface of the cuticle covering the eye (Fig. 1c,r–u), exposing clear exocones, very similar to those of xiphosurans (Fig. 1n–p). In the detached part of the fossil they are indicated by tapering cavities (GIK 186a, Fig. 1q). These exocones, like their negatives, remain quite separated from each other, rather than being densely packed as are the facets of a honey bee, hornet, a dragon fly or of many trilobites (Fig. 1f–h,l,m). Our results clearly show that the typical shape of exocones in the eurypterid eye is similar to those of *Limulus* (Fig. 1n–u) and these exocones may have functioned in the same way as lens cylinders.

### Mode of preservation and receptor system

The second question that has to be considered is the construction of the receptor system. This is a much more difficult problem, because the relics of soft (labile) tissues, such as nervous or sensory cells, are very rarely found in the fossil record, and then only as a result of special kinds of preservation, such as phosphatisation, or at particular Konservat-Lagerstätten *sensu* Seilacher *et al*.^66^. Such exceptional preservation of soft tissue has not hitherto been known from eurypterid fossils. The grain size of the host sediment, in most cases a clayey siltstone, lies between silt (0.002–0.063 mm) and clay (<0.002 mm), in the range that would preserve the traces of cellular structures very well because the size of the receptor cells is larger than the grain-size. There is another adverse effect: In many eurypterid fossils no facets can be observed, the eyes appear as covered by relicts of a pellucid membrane, probably the cuticle^21^. Thirdly, most eurypterid fossils are moults^13^ and it is only in dead individuals (carcasses) that preservation of the internal structure of fossil eyes can be expected.

### Sublensar rosettes

Only a few examples of microstructures of visual systems in fossils have been reported so far^39,67–69^. Thus the following analysis should be understood as just a first attempt to explore the sensory concept and likely in consequence the relationship of the eurypterids to other arthropod groups based on the comparison of visual systems. This may provide insights into the evolution of compound eye systems in general.

All specimens illustrated here (Fig. 1c–e) reveal a typical pattern of an ommatidium in cross section, which results from an overhead view, when the overlying parts of the eye have been lost during the course of time. In most of these a zone of former pigment (cells) can be distinguished round the periphery, sometimes even having a dark appearance, possibly due to relicts of the carbon formerly contained in the pigment cells. More centrally traces of sensory cells can be made out, each arranged like a rosette and of different number among the systems (5–9 cells, Fig. 2a–d,f,k–u). In diameter of ~70 µm the units are very similar in size to the receptor cells of adult *Limulus* (~70 µm^70,71^). The most important part, the centre, repeats the pattern mentioned above in describing the *Limulus* rhabdomeric structure. Though the fine individual microvillar structures themselves have not been preserved, we find a comparable wide dark ring with a more or less circular bright structure in the centre. It is quite remarkable to find such a central structure in a fossilised rhabdom, but very clearly it is evident in Fig. 2a–f,o,p–r, and indicated as a central dot inside of the rhabdom in Fig. 2s–u.

There is a good case for assuming (see below) that the dark ring represents the relics of the rhabdomeric/microvillar arrays, the bright centre the dendrite of an eccentric cell. This is a recurrent pattern in all examples we found, illustrated in Fig. 2. It strongly suggests that the eurypterid system is of the xiphosuran type.

## Discussion

The results above clearly show that in terms of their ordering, the lens cylinders (exocones) and the internal structure of the underlying visual unit (variable number of receptor cells, very probably the existence of an eccentric cell as an element of the ommatidium), the eurypterid eyes are almost identical to xiphosuran compound eyes.

While in highly resolving mandibulate apposition compound eyes the facets are ordered normally in a hexagonal pattern, in *Limulus* the ommatidia are irregularely positioned^50^. The xiphosurans investigated here show a regular, squared pattern of the facets. Squared arrangements of lenses are typical for superposition eyes of mandibulates, namely among decapod crustaceans^54^. To find a squared pattern in *J. rhenaniae* indicates an autonomous character, perhaps suggesting a specialised visual system.

There is an important issue to discuss at this juncture, the relatively enormous size of the receptors in the compound eyes of *Jaekelopterus rhenaniae*. Figure 2m–u show the conspicuously large relics of the eurypterid receptor cells, which reach sizes of ~70 µm. In human retinas, the receptor size lies at the limit of any physically possible dimension - at about 1 µm, the lower limit of arthropod receptor diameters consequently also lies at 1–2 µm, while the diameter of normal ommatidia as a whole achieves between 5–50 µm^72^. The diameter of rhabdoms often reaches between 1.5–3.5 µm^43^, consequently the size of a normal receptor cell is smaller or around 10 µm. In particular, the photoreceptors of *Limulus*, however, are among the largest in the animal kingdom^73^. *Limulus*, as the benchmark needed here, the size of the rhabdom lies at about 60 µm (diameter^69^), equal to those as found in *J. rhenaniae* (~60 µm, Fig. 2o), the size of the entire ommatidium at 170–~250 µm^73,74^, are identical to the diameters of our material (~250 µm, all examples of Fig. 2). Thus, the sizes of the ommatidia of *J. rhenaniae* match absolutely those of *Limulus*. Consequently, the size of these photoreceptors is the ~70-fold of the human or common arthropodan receptor size.

At this point some more references may be cited to support this important issue. Firstly, the present classic reference about the morphology of the *Limulus* compound eye is that of Fahrenbach 1969, p. 252^70^. He describes how the body of the ommatidium is formed by primary receptor cells with 70 µm in diameter, the retinula. They are grouped like orange slices about the central tapering dendrite of the secondary receptor, the eccentric, cell. The eccentric cell, a modified receptor cell, also measures about 70 µm. Fahrenbach notes that the number of retinula cells per ommatidium averages between 10 and 13 for different individuals, the observed range covering 4 to 20 cells. (^70^, p. 252), in the eurypterid we find 5 to 9 cells. These descriptions of the *Limulus*-eye were confirmed by other authors, such as Batelle 2016, p. 810^75^: [photoreceptor cells: about 150 µm long and 70 µm wide in adults, furthermore^74^, p. 261^76^, p. 416, Fig. 2, and many others.] The enormous size of the *Limulus* photoreceptors gave rise to the first electrophysiological intracellular recordings in the nineteen-sixties - seventies (e.g.^77,78^, and other research) discovering the ionic mechansims of transduction and the discrete waves (quantum bumps) as responses to single photons (^79–82^, and others). As a whole, these investigations, based on the exceptionally large photoreceptors of *Limulus* established an important part of modern electrophysiology.

Since Exner in 1891^50^ described the fascinating physical properties of the lens cylinders in the compound eye of the xiphosuran *Limulus*, this system has been subject of intense research, morphologically and physically (for an overview see^52,83,84^). It is now evident that this eye is, not just in consideration of its dioptric apparatus, very different from any tetraconate compound eye, such as that of a bee or dragonfly, but also in its entire morphology. In the eye of the horseshoe crab the receptor cells are coupled directly in the rhabdom, namely by the central dendrite of the eccentric cell. The function of the eccentric cells, probably among others, is to enhance contrasts and edge-perception, important especially for an organism active at low light conditions and in the scattered light conditions of the water column. The similarly structured rhabdom in the eurypterid *Jaekelopterus rhenaniae* investigated here, allows to assume that the same function already existed at least since the Lower Devonian.

The earliest horseshoe crab-fossils come from the Early Ordovician (more than 470 Mya old)^47^, and the family Limulidae, comprising all extant species, dates back to the Triassic, about 240 Mya^85^. Today there exist four species of three genera. Many of the common characteristics of eurypterids and xiphosurans have been considered to be symplesiomorphic or convergent due to a shared aquatic life style. As mentioned before, the eyes of modern arachnids, however, are not compound eyes, but single-lens eyes, and probably developed by partition of compound eyes and fusion of their ommatidia^55–57^. They must be understood as highly derived systems, while their ancestral structure, for example the existence of eccentric cells, can no longer be perceived. As mentioned above, Palaeozoic and Mesozoic scorpions, also possessed compound eyes, in outer appearance not dissimilar to eurypterid eyes^55^. Very probably, according to the results presented here, their internal compound eye-structures also may have been similar.

Most recently Fleming *et al*.^86^ analysed a large-scale data set of ecdysozoan opsins; comparing this with morphological analyses of key Cambrian fossils with preserved eye structures. They found that multi opsin vision evolved in a period of 35–71 million years through a series of gene duplications. They show that while Chelicerata have 4 opsins, Pancrustacea (crustaceans and hexapods) already possess 5^85^. Consequently this may indicate an earlier origin of the chelicerate visual system, because to develop the fifth opsine takes time (principle of genetic clocks). Bitsch and Bitsch^87^, referring to Fahrenbach^51,70,88,89^ considered the eccentric cell to be autapomorphic to xiphosurans (ref. ^87^, p. 188). If, however, the eurypterids possessed an eccentric cell as well, this structure may be plesiomorphic to Chelicerata. Fahrenbach described that double eccentric cells are not uncommon in *Limulus* (ref. ^70^, p. 452). A single eccentric cell, however, is typical, and a further reduction during evolution in the context of changing ecological constrains and along phylogeny can be imagined easily. This element may not belong to the oldest form of any compound eye, its form and occurrence lies still in the dark.

Functionally, with several thousands of facets (>3545 facets/eyes and >747.235 mm² eye area)^22^, and probably being equipped with a contrast enhancing neuronal system due to the eccentric cells, the giant sea scorpion *Jaekelopterus rhenaniae* from the Early Devonian of Germany had a very effective visual capacity for high acuity and sensitivity, excellently adapted to an efficient, visually guided, aquatic predatory life-style.

In summary our results suggest a close similarity between the xiphosuran and the eurypterid eye, confirming the basal phylogenetic connection between both forms and lending support for a close relationship as indicated by some phylogenetic analyses (e.g.^90^). Convergent eye evolution, generating more or less identical sophisticated visual systems in xiphosurans and eurypterids as found here, seems rather improbable. This means, that the visual system equipped with lens cylinders and an eccentric cell with all its functions, is very basal. If so, this furthermore implies that the Palaeozoic arachnid visual system also possessed an eccentric cell. This primordial arachnid eye may then have given rise to the eyes of modern arachnids in the course of terrestrialisation.

This is also supported by the notion that the oldest known arachnids, the Silurian scorpions, had compound lateral eyes^58,91–93^, while the lateral eyes of extant crown group scorpions consist of two to five pairs of lateral lenses^86^. Perhaps in relation to a ground-dwelling terrestrial life-style extant arachnids mostly seem to rely on non-visually sensory systems such as mechanoreceptors (trichobothria, pectines and highly sensitive setae) or chemosensory systems, which seem to be metabolically much less expensive than visual systems^94,95^; so eyes may become reduced or disappear. One may remember here, that many of the arthropods appearing with the Cambrian explosion, were benthic, requiring a visual system quite sensitive to light, and taking advantage of any contrast enhancing system such as lateral inhibition, which is tied up to the eccentric cells. Even if the evidence of the lens cylinders in the eurypterid eye, as strong indications of the existence of eccentric cells, represented by their dendrites in the centres of the rhabdoms, is given here, future findings of better preserved material may confirm these findings by the use of synchrotron radiation or computer tomography. Appropriate material is, to our knowledge, still lacking, and thus the application of these methods is not possible, perhaps will never be, and consequently the evidence given here may remain all that is attainable at present.

## Material and Methods

The macrophotograps of Figs. 1c–l,n–u, 2q–u were taken with a Keyence digital microscope **(**VHX-700F, objective VH-Z20T and at the Institute of Biology Education (Zoology), University of Cologne. The specimens were not submerged in isopropanol in order to retain the contrasts of the edges. The pictures were processed and arranged to figures with Adobe Photoshop CS3 and Adobe Photoshop Elements.

The macrophotographs of Fig. 2a–d were taken with the specimens submerged in isopropanol using a Canon EOS 600D SLR camera equipped with a Canon MP-E 65 mm macro lens. Free image stacking software (CombineZM by Alan Hadley) was then employed to produce composites with enhanced depth of field using photographs with differing focal planes. These were processed and arranged into figures using Adobe Photoshop CS3.

The SEM photographs were taken at the Biocentre Cologne, (FEI -Quanta FEG 250, Thermo Fischer Scientific).

The eurypterid eyes figured in this contribution are stored in the Geological Institute of the University of Cologne, leg. E. Evangelou (Figs. 1c–e,q–u, 2). They were collected from Siegenian (possibly lowermost Emsian in terms of the international stratigraphic frame) strata of the Jaeger Quarry near Frielingshausen/Bergisches Land, Germany.
